# Association of plasma high-density lipoprotein cholesterol level with risk of stress urinary incontinence in women: a retrospective study

**DOI:** 10.1186/s12944-024-02137-6

**Published:** 2024-06-07

**Authors:** Wenning Xu, Baojia Zheng, Lili Su, Yali Xiang

**Affiliations:** Health Management Center, The Fifth Affiliated Hospital of Sun Yat-sen University, Zhuhai, China

**Keywords:** Stress urinary incontinence, High-density lipoprotein cholesterol, Risk factor, Lipid

## Abstract

**Background:**

Studies have found that high density lipoprotein cholesterol (HDL-C) levels are linked to a variety of diseases. However, evidence for the relationship between stress urinary incontinence (SUI) and HDL-C remain limited.

**Methods:**

590 eligible women were enrolled. Basic characteristic, gynecological examinations and blood sampling were collected. The examination of the possible link between HDL-C and SUI was done using univariate and multivariate logistic regression. Feature importance ranking and Receiver operating characteristic (ROC) curves were performed to further evaluate the association between HDL-C and SUI in women.

**Results:**

A significant association was found between HDL-C and SUI in women, revealing higher HDL-C levels were related to a lower risk of SUI (OR 0.238; 95%CI: 0.091–0.623; *P* < 0.01) after adjustment for potential key confounders. The AUC for the SUI predicted by the combined HDL-C was 0.845 (95%CI: 0.798–0.891, *P* < 0.001). The feature importance ranking revealed that vaginal delivery, HDL-C were the top two important factors.

**Conclusions:**

HDL-C levels were correlated with the development of SUI. In addition to physical and surgical treatments, HDL-C may offer the possibility of potential targeted treatment and prevention of SUI afterwards.

## Background

Approximately 43–349 million Chinese women experience urinary incontinence (UI), with stress urinary incontinence (SUI) being the most prevalent type [1]. Urine leakage results from SUI (severe abdominal pressure) when intraabdominal pressure surpasses urethral pressure [2], which has adverse impact on women’s daily activities and socialization. Urine leakage due to SUI is also related to depression, work impairment, and sexual dysfunction [3]. Even though the treatments for SUI are costly, the clinical effect is still not clear [4]. According to statistics, 10–40% of women experience severe stress incontinence [5]. When a woman reaches the age of 40 or older, the frequency of SUI rises with age, peaking at 50% [6]. Physical therapy, medication intervention, and surgical treatment are currently used to treat SUI [7, 8], while the control of risk factors and lifestyle changes also significantly improve patients’ symptoms.

Most evidence suggests that menopause, obesity, and vaginal delivery play a role in the development of SUI [9]. Additionally, the pathophysiological variation in SUI has gradually gained increasing attention. The primary lipoprotein that carries cholesterol from the peripheral back to the liver for elimination is high-density lipoprotein cholesterol (HDL-C). HDL has previously piqued interest due to its potential to prevent atherosclerotic cardiovascular disease. Moreover, the decreased HDL-C levels are also linked to increased risks of death during sepsis, infectious diseases, chronic kidney disease, and diabetes mellitus [10].

A previous study revealed that there was no link between urinary incontinence and cholesterol levels [11]. But women with SUI had lower HDL levels than control women, according to Dursun et al [12]. Currently, the association between HDL and SUI remains controversial, with limited evidence. The purpose of this study was to assess the association between HDL and SUI and offer insights into the management and treatment of patients with SUI.

## Methods

### Study design and participants

This retrospective study was carried out at the Health Management Center of the Fifth Affiliated Hospital of Sun Yat-sen University between May 2021 and July 2022. The eligibility criteria were as follows: (1) was female and (2) was willing to voluntarily participate in this clinical study. The exclusion criteria included: (1) had cognitive impairment or could not understand and finish the survey; (2) were pregnant or gestating females; (3) had psychiatric disorders; (4) had neurological disorders like multiple sclerosis or Parkinson’s disease; (5) had malignant tumours; and (6) had premature menopause due to certain medical reasons. Our study, which was approved by the Research Ethics Committee of the Fifth Affiliated Hospital of Sun Yat-sen University, Zhuhai, China (No. 2021-K32-1), was part of a randomized controlled trial to improve SUI in women (ChiCTR:2,100,047,215). Participants were required to provide written informed consent before enrolling in the study.

### SUI definition and screening

Urine overflow occurred with varied degrees of elevated abdominal pressure (by lifting, coughing, or activity), and SUI is diagnosed based on common symptoms [13]. The participants were asked “Do you experience urinary leakage with coughing, sneezing, laughing, or exercising?” by a gynaecologist and completed the incontinence questionnaire-urinary incontinence short form (ICI-Q-SF) for SUI screening. The four items on the ICI-Q-SF include frequency of volume of leakage, urine incontinence, self-diagnostic items, and overall impact of urine incontinence. The ICI-Q-SF is a reliable tool for evaluating symptoms of UI and their influence on patients` life quality [14]. The total score headed from 0 to 21, with lower scores indicating better conditions. The ICI-Q-SF score was 0 in the absence of urine incontinence [15]. Patients with an ICI-Q-SF score greater than or equal to 1 who also had typical symptoms of SUI were classified into the SUI group.

### Data collection

The eligible women were approached by a gynaecologist to complete the SUI screening and gynaecological examination. Then, specialized and trained nurses drew blood samples and administered a pretested questionnaire to collect demographic, delivery and pelvic status information. The following information was recorded: sociodemographic characteristics [including age, educational level, occupation, and annual income]; individual characteristics [body mass index (BMI), blood pressure (BP), smoking status, and history of medication for chronic disease]; delivery and pelvic status (including age at first delivery, vaginal delivery, history of pelvic surgery, and vaginitis); and physiological indicators [including albumin (ALB), haemoglobin (Hb), total protein (TP), low-density lipoprotein cholesterol (LDL-C), high-density lipoprotein cholesterol (HDL-C), triglyceride (TG), and total cholesterol (TC), including r-glutamyl transpeptidase (GGT), glutathione transaminase (ALT), glutamic oxalacetic transaminase (AST), creatinine (Cr), uric acid (UA), neutrophilic granulocyte (NEUT), lymphocyte (LYM), white blood cell (WBC), and mononuclear cell (MO)].

After the participants filled out the relevant questionnaires, two researchers reviewed the data to determine the validity and looked for any missing items. If there were too many missing data, the researchers conducted a telephone consultation with the participants.

### Statistical analysis

All data analysis was completed by SPSS software (version 22.0). Proportional distributions and frequency were used to represent categorical variables, while the mean ± standard deviation was used to express continuous variables. The chi-square test or Fisher’s exact probability test were used to examine the sociodemographic traits of the patients in the SUI and non-SUI groups (Table 1). To look into the independent relationships between each factor (Table 2) and SUI development, binary logistic regression was employed. The significant sociodemographic characteristics, including age and occupation, were included as covariates in each logistic regression equation for adjustment. Odds ratios (ORs) and 95% confidence intervals (CIs) were calculated in the models. The multivariable logistic analysis (Table 3) was adjusted for all the risk factor variables included in the full model and refined by enter regression. Receiver operating characteristic (ROC) curves were used to analyse the efficacy of potentially modifiable risk factors in predicting SUI, and the area under the curve (AUC) was calculated. The feature importance was ranked by measuring the contribution (x) of the standardized regression coefficients of each significant variable in the model [16]. The level of significance was set at *P* < 0.05.

**Table 1:** The sociodemographic characteristics of enrolled participants

Variables		non-SUI(*N* = 273)		SUI(*N* = 317)		χ2	*P* value
		N	%	N	%		
*Age*	*< 35*	*178*	*65.2*	*73*	*23.0*	*106.726*	*< 0.001*
	*≥ 35*	*95*	*34.8*	*244*	*77.0*		
Educational level	High school and below	51	18.7	80	25.2	3.649	0.056
	University or higher	222	81.3	237	74.8		
*Occupation*	*Unemployed*	*23*	*8.4*	*56*	*17.7*	*14.146*	*< 0.001*
	*Intellectual laborer*	*212*	*77.7*	*205*	*64.7*		
	*Manual laborer*	*38*	*13.9*	*56*	*17.7*		
Annual income(¥)	< 100,000	137	50.2	177	55.8	1.883	0.170
	≥ 100,000	136	49.8	140	44.2		

**Table 2:** Univariable analysis for the association of SUI with individual characteristics, delivery and pelvic status

Variables		non-SUI (*N* = 273)		SUI(*N* = 317)		Adjust OR(95%CI)	*P* value
		N	%	N	%		
Individual characteristics							
*Body mass index (BMI)*	*<18.5*	*53*	*19.4*	*12*	*3.8*	*Ref*	
	*18.5–23.9*	*182*	*66.7*	*211*	*66.6*	*4.043(2.011–8.132)*	*<0.001*
	*≥ 24*	*38*	*13.9*	*94*	*29.7*	*6.858(3.134–15.003)*	*<0.001*
Blood pressure	Normal	262	96	282	89.0	Ref	
	Low	4	1.5	9	2.8	0.483(0.132–1.770)	0.272
	High	7	2.6	26	8.2	1.029(0.216–4.912)	0.971
Smoking	No	270	98.9	316	99.7	Ref	
	Yes	3	1.1	1	0.3	0.624(0.054–7.220)	0.706
*History of medication*	*No*	*256*	*93.8*	*287*	*90.5*	*Ref*	
	*Yes*	*17*	*6.2*	*30*	*9.5*	*2.355(1.190–4.663)*	*0.014*
Delivery and pelvic status							
Age of first delivery(years)							
	< 30	211	77.3	247	77.8	Ref	
	≥ 30	62	22.7	70	22.2	0.913(0.544–1.531)	0.729
*Vaginal delivery*	*0 time*	*194*	*71.7*	*53*	*16.7*	*Ref*	
	*1 time*	*47*	*17.2*	*137*	*43.2*	*7.661(4.760-12.328)*	*<0.001*
	*≥ 2 times*	*32*	*11.7*	*127*	*40.1*	*10.158(6.024–17.128)*	*<0.001*
*History of pelvic surgery*	*No*	*269*	*98.5*	*293*	*92.4*	*Ref*	
	*Yes*	*4*	*1.5*	*24*	*7.6*	*4.502(1.446–14.019)_*	*0.009*
*Vaginitis*	*No*	*244*	*89.3*	*250*	*78.9*	*Ref*	
	*Yes*	*29*	*10.7*	*67*	*21.1*	*2.148(1.284–3.594)*	*0.004*

Adjusted by age and occupation

**Table 3:** Univariable analysis for the association of SUI with physiological indicators

Variables	non-SUI (*N* = 273)	SUI (*N* = 317)	Adjust OR(95%CI)	*P* value
	Mean ± SD	Mean ± SD		
TG (mmol/L)	1.00 ± 0.77	1.11 ± 0.62	1.088(0.795–1.489)	0.597
TC (mmol/L)	4.86 ± 0.95	4.86 ± 0.89	0.898(0.727–1.109)	0.318
*HDL-C (mmol/L)*	*1.62 ± 0.34*	*1.47 ± 0.29*	*0.281(0.147–0.538)*	*<0.001*
LDL-C (mmol/L)	2.60 ± 0.83	2.73 ± 0.80	1.087(0.847–1.385)	0.512
*TP (g/L)*	*75.62 ± 3.95*	*73.36 ± 3.84*	*0.867(0.816–0.921)*	*<0.001*
ALB (g/L)	47.43 ± 2.53	46.60 ± 2.45	0.901(0.822–0.988)	0.026
*Hb(g/L)*	*128.33 ± 11.92*	*131.88 ± 11.75*	*1.031(1.015–1.047)*	*<0.001*
*GGT (U/L)*	*14.70 ± 13.21*	*21.55 ± 35.51*	*1.019(1.001–1.037)*	*0.039*
*ALT (U/L)*	*12.50 ± 12.67*	*17.32 ± 13.93*	*1.021(1.003–1.040)*	*0.025*
AST (U/L)	18.48 ± 13.50	18.03 ± 15.19	0.999(0.993–1.004)	0.620
*Cr (µmoI/L)*	*62.21 ± 9.39*	*60.61 ± 10.16*	*0.981(0.963-1.000)*	*0.047*
UA (µmol/L)	289.26 ± 65.31	285.92 ± 63.25	0.999(0.997–1.002)	0.722
WBC (*10^9^/L)	6.24 ± 1.60	6.12 ± 1.49	0.988(0.876–1.114)	0.841
NEUT (*10^9^/L)	3.50 ± 1.18	3.52 ± 1.19	1.022(0.875–1.195)	0.781
LYM (*10^9^/L)	2.15 ± 0.58	2.03 ± 0.53	0.899(0.643–1.257)	0.533
MO (*10 ^*12*^ */L)*	*0.40 ± 0.13*	*0.38 ± 0.10*	*0.168(0.035–0.794)*	*0.024*

Adjusted by age and occupation

TG, Triglyceride; TC, Total Cholesterol; HDL-C, High-density lipoprotein cholesterol; LDL-C, Low-density lipoprotein cholesterol; TP, Total protein; ALB, Albumin; GGT, r-glutamyl transpeptidase; ALT, Glutathione transaminase; AST, Glutamic oxalacetic transaminase; Cr, Creatinine; BU, Urea; UA, uric acid; PLT, Platelets; WBC, White Blood Cell; NEUT, neutrophilic granulocyte; LYM, Lymphocytes; Mo, Mononuclear cells. Hb, hemoglobin

## Results

### Sociodemographic characteristics of the SUI group and non-SUI group

590 participants were eligible and enrolled to finish questionnaires on basic characteristics, gynaecological examinations and blood sampling for collecting information. Based on symptom confirmation and the ICI-Q-SF assessment, 273 women were in the non-SUI group, and 317 were in patients` group. The significant differences were found in ages ranging from 20 to 68 years (*P* < 0.001) and occupation (*P* < 0.001) between the SUI group and non-SUI group, whereas no significant differences in education level or annual income between the two groups in Table 1.

### Univariate analysis

**T**he results of the univariable analysis for the association of SUI with individual characteristics, delivery and pelvic status shown in Table 2. The risk was greatly increased for women with a higher BMI (OR: 6.858; 95%CI: 3.134–15.003). Obviously, the prominent risk factor for SUI was vaginal delivery, and women who gave birth vaginally twice (OR: 10.158; 95%CI: 6.024–17.128) had a greater increase in risk than those who gave birth once (OR: 7.661; 95%CI: 4.760-12.328). Moreover, SUI was also associated with a history of pelvic surgery (OR: 4.502; 95%CI: 1.446–14.019), a history of medication (OR: 2.355; 95%CI: 1.190–4.663) and vaginitis (OR: 2.148; 95%CI: 1.284–3.594). The correlations between potential physiological indicators and the risk of SUI in women were shown in Table 3. The mean HDL-C level was 1.62 mmol/L (SD 0.34) for the non-SUI group and 1.47 mmol/L (SD 0.29) for the SUI group, suggesting that a lower HDL-C level was related to a higher risk of SUI (OR: 0.281; 95%CI: 0.147–0.538). Table 3 showed strong correlations between the following parameters and SUI adjusted by age and occupation: TP (OR: 0.867; 95%CI: 0.816–0.921), Hb (OR: 1.031; 95%CI: 1.015–1.047), GGT (OR: 1.019; 95%CI: 1.001–1.037), ALT (OR: 1.021; 95%CI: 1.003–1.040), Cr (OR: 0.981; 95%CI: 0.963-1.000) and MO (OR: 0.168; 95%CI: 0.035–0.794). The other physiological indicators were not found the significantly difference between the two groups.

### Multivariable modifiable risk factor analysis

Three models were constructed to adjust the relationships between the variables step by step, as shown in Table 4. Model 1 incorporated only individual characteristics; Model 2 incorporated individual characteristics plus delivery and pelvic status; and Model 3 incorporated individual characteristics, delivery and pelvic status, and physiologic indicators. HDL-C continued to be an independent factor, as well as a positive indicator against SUI (OR: 0.238; 95%CI: 0.091–0.623; *P* < 0.01), after adjustment for age, occupation, BMI, history of medication, vaginal delivery, history of pelvic surgery, vaginitis, TP, ALB, Hb, GGT, ALT, Cr and MO.

**Table 4:** Multivariable association between potential risk factors and SUI

Variables	Units	Adjusted OR(95%CI)	Variables	Units	Adjusted OR(95%CI)	Variables	Units	Adjusted OR(95%CI)
Model 1: individual characteristics			Model 2: individual characteristics + delivery and pelvic status			Model 3: individual characteristics + delivery and pelvic status + physiological indicators		
BMI(1)	18.5–23.9	3.002(1.415–6.370)**	BMI(1)	18.5–23.9	3.169(1.407–7.134)**	BMI(1)	18.5–23.9	3.382(1.181–9.688)*
BMI(2)	≥ 24	4.291(1.820–10.120)***	BMI(2)	≥ 24	4.747(1.875–12.020)**	BMI(2)	≥ 24	4.518(1.307–15.620)*
History of medication	Yes	2.139(1.071–4.271)*	History of medication	Yes	2.462(1.133–5.354)*	History of medication	Yes	3.226(1.087–9.578)*
			Vaginal delivery(1)	1 times	5.648(3.209–9.941)***	Vaginal delivery(1)	1 times	4.367(2.108–9.045)***
			Vaginal delivery(2)	≥ 2 times	8.646(4.732–15.799)***	Vaginal delivery(2)	≥ 2 times	8.348(3.738–18.643)***
			History of pelvic surgery	Yes	2.761(0.788–9.882)	History of pelvic surgery	Yes	1.475(0.257–8.452)
			Vaginitis	Yes	1.700(0.922–3.134)	Vaginitis	Yes	2.208(0.986–4.946)
						HDL-C		0.238(0.091–0.623)**
						TP		0.914(0.834–1.001)
						ALB		1.069(0.921–1.242)
						Hb		1.021(0.995–1.048)
						GGT		1.006(0.990–1.022)
						ALT		1.013(0.991–1.035)
						Cr		1.001(0.970–1.033)
						MO		0.156(0.012–2.045)

Adjusted by age and occupation. *,*P* < 0.05; **,*P* < 0.01; ***P < 0.001. HDL-C, High-density lipoprotein cholesterol; TP, Total protein; ALB, Albumin; Hb, hemoglobin; GGT, r-glutamyl transpeptidase; ALT, Glutathione transaminase; Cr, Creatinine; MO, Mononuclear cells

### The association of HDL-C with SUI

**Fig. 1:**
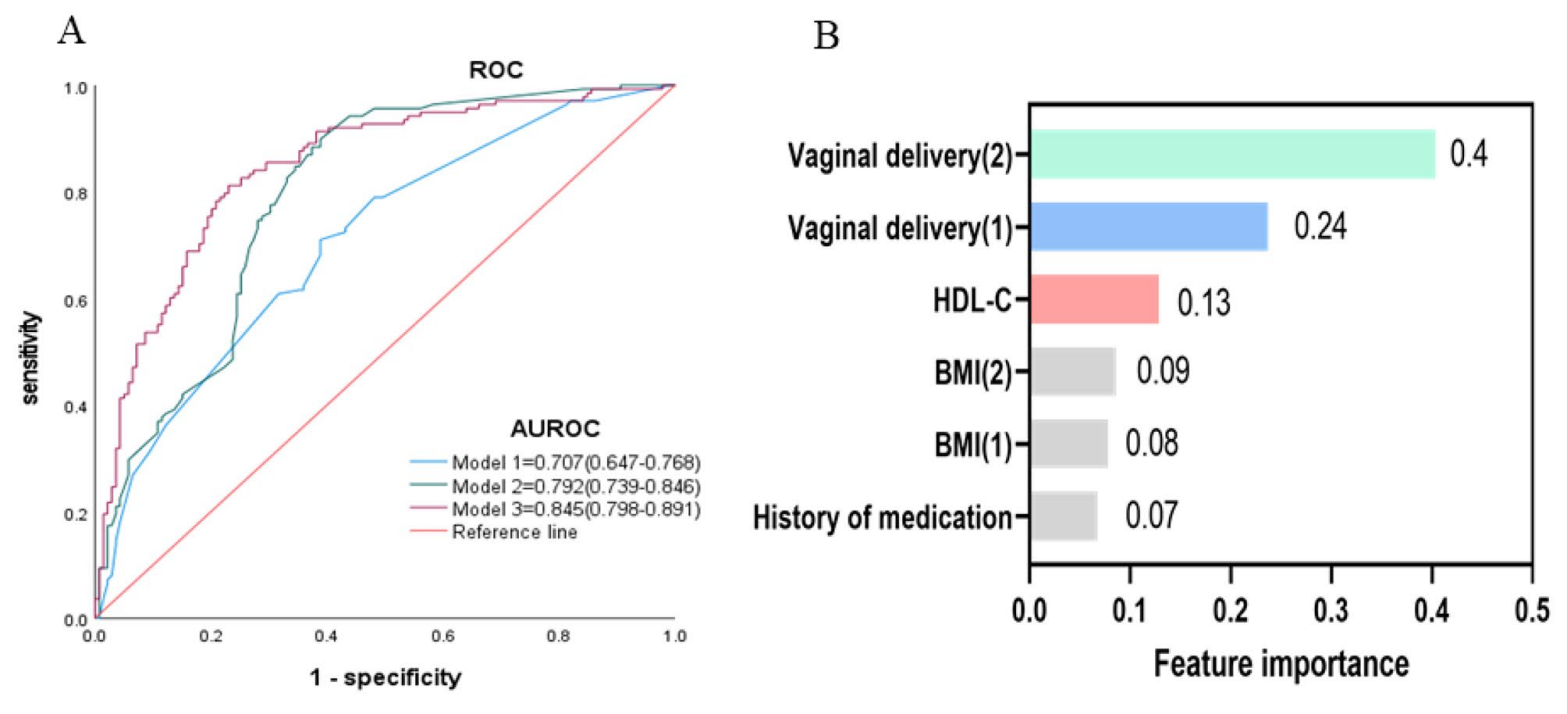
The effect of HDL-C associated with stress urinary incontinence in women.(**A**) Receiver operating curves (ROC) of model 1, model 2 and model 3 in predicting the development of SUI. (**B**) Feature importance ranking of significant variables in logistic regression of model 3

The ROC curve was used to analyse the efficacy of the three models described above in predicting the development of SUI. The area under the receiver operating characteristic curve (AUROC) of Model 3 was shown in Fig. 1A, which included BMI, history of medication, vaginal delivery and HDL-C, reached 0.845 (95% CI: 0.798–0.891; *P* < 0.001). To explore the contribution of HDL-C to the model, standardized regression coefficients were used to rank the importance of the top features, as described in Fig. 1B. As a result, HDL-C ranked second only to vaginal delivery as the top variable, suggesting its vital role in the onset of SUI. HDL-C was used as a categorical variable with three subgroups based on the 33.3rd percentile and 66.66th percentile of the queue to further clarify the relationship between SUI and HDL-C. The lower the HDL-C level was linked to the greater risk of developing SUI (OR: 0.521; 95%CI: 0.284–0.957) with *P* value of 0.035 after adjusting for age, occupation, BMI, history of medication, and vaginal delivery (Table 5),.

**Table 5:** Association between the groups of HDL-C and SUI in women

Variables	Univariable		Multivariable	
	OR(95%CI)	*P* value	OR(95%CI)	*P* value
Group 1 (< 1.39)	Ref		Ref	
Group 2 (1.39–1.64)	0.533(0.322–0.882)	0.014	0.535(0.286–1.002)	0.051
Group 3 (≥ 1.64)	0.402(0.247–0.654)	< 0.001	0.521(0.284–0.957)	0.035

Univarible analysis was adjusted by age and occupation. Multivariable regression analysis was adjusted by age, occupation, BMI, history of medication, vaginal delivery

## Discussion

Despite the fact that SUI is a prevalent issue, only 25% of patients seek or receive treatment [17]. Identification of potential risk factors and early intervention are essential for improving SUI. The development of SUI has been linked to a number of risk factors, such as age, BMI, and past childbirth [18]. However, the pathophysiological aspects of SUI have not been thoroughly studied, and further research is necessary to explore potential intrinsic treatments and management. HDL is increasingly targeted for the therapy of some disorders because of its unique function [19]. The current evidence of the relationship between HDL and SUI is limited and ambiguous. Therefore, the study aimed to examine the relationship after adjusting for a range of correlates of SUI.

After adjusting for age and occupation, vaginal birth and BMI were found to be the most robust markers of SUI in the study. However, after controlling for a number of covariates, greater HDL-C levels were substantially linked to a decreased risk of SUI. Moreover, HDL-C was employed as a categorical variable, and a decreased probability of SUI occurrence was linked to a larger level of HDL-C. There was no relation between cholesterol levels and urinary incontinence, according to a report form Ebbesen et al. In 2009 [11]. Other previous surveys in primiparas, perimenopausal and postmenopausal old women also revealed that no statistically significant difference in HDL-C between two groups [20–22]. However, an elevated incidence of metabolic syndrome or a high visceral fat index in patients with SUI and decreased HDL-C levels were also found [12, 23]. Although there are discrepancies in the results of existing studies on HDL-C and SUI, these findings are still valuable as they support the use of this parameter for improving the symptoms of SUI. In the study, HDL-C was separated into continuous and categorical variables for logistic analysis, and ROC curves and feature importance rankings were combined to clarify the link between HDL-C and SUI by enhancing the reliability of the evidence.

The correlation between HDL-C and SUI has been reported to involve several potential mechanisms. Large HDL-C levels are found to be inversely correlated with visceral fat area [24]. Higher intra-abdominal pressure and a higher risk of SUI result from greater visceral fat [25]. Second, HDLs promote the integrity and function of the endothelium barrier, inhibit the inflammatory effects of innate and adaptive immune cells, and induce angiogenesis [26, 27]. In addition, increasing levels of HDL-C decrease insulin resistance, which has been linked to the progression of inflammation, resulting in urinary incontinence (UI) [28]. Conversely, inverse relationships between HDL-C and central obesity were discovered [29], and elevated intra-abdominal pressure brought on by high levels of central obesity, insulin resistance, and oxidative stress leading to pelvic floor vascular injury with sphincter and forced urinary muscle dysfunction are some of the potential mechanisms that may cause UI [30]. It has been observed that in healthy young to middle-aged individuals without cardiometabolic disease, HDL-C and apolipoprotein A-I improve skeletal muscle [31]. Low HDL-C levels are linked to SUI, which may be explained by the correlation between trunk muscle mass and the status and severity of SUI [32]. Subjects with a normal diet of saturated fat had a mean blood HDL-C content that was lower than that of subjects with a normal intake of saturated fat [33]. However, increased saturated fat intake can enhance autonomic nervous system activity, leading to less urinary tract symptoms and an overactive bladder, contributing to SUI [34].

### Study strengths and limitations

There were several strengths of the present study. Qualitative and quantitative data were comprehensively collected in this study to control for the influence of covariates on SUI as much as possible to verify the relationship between HDL-C and SUI. Additionally, SUI screening and gynaecological examinations were performed by a team of experienced gynaecologists to ensure that the enrolled participants had a high probability of experiencing SUI. However, there were still some limitations. This study was retrospective and single-center. Due to the limited sample size, an unbiased matching design was not used, and age and occupation were used as covariables in each regression equation for adjustment. Even though this study collected a lot of data on demographics, delivery-pelvic-related factors, and blood markers, the observational study design still limited our capacity to account for confounding variables including unmeasured confounders.

## Conclusion

These findings provide light on the connection between HDL-C and SUI and point to potential opportunities for SUI treatment in women. Clinically, the finding of the association between HDL-C and SUI facilitates early clinical identification of high-risk patients, as well as providing potential therapeutic targets and interventions to improve symptoms in patients with SUI.

## Data Availability

No datasets were generated or analysed during the current study.

## Abbreviations

ICI-Q-SF: Incontinence questionnaire-urinary incontinence short form
SUI: Stress urinary incontinence
UI: Urinary incontinence
BP: Blood pressure
ALB: Albumin
TP: Total protein
TG: Triglyceride
LDL-C: Low-density lipoprotein cholesterol
HDL-C: High-density lipoprotein cholesterol
TC: Total cholesterol
GGT: r-glutamyl transpeptidase
ALT: Glutathione transaminase
AST: Glutamic oxalacetic transaminase
Cr: Creatinine
UA: Uric acid
WBC: White blood cell
NEUT: Neutrophilic granulocyte
LYM: Lymphocytes
MO: Monocyte
ROC: Receiver operating characteristic
AUC: Area under the curve
